# Administrative criteria in the relapse of depressive disorder: a critical appraisal

**DOI:** 10.3389/fpsyt.2026.1796386

**Published:** 2026-08-18

**Authors:** Sara Bernal-Cueto, Fanny Cegla-Schvartzman, Enrique Baca-García

**Affiliations:** 1 Department of Psychiatry, Jimenez Diaz Foundation Hospital, Madrid, Spain; 2 Instituto de Investigación Sanitario de la Fundación Jiménez Díaz, Madrid, Spain; 3 Department of Psychiatry, Universidad Autónoma de Madrid, Madrid, Spain; 4 Department of Psychiatry, Rey Juan Carlos University Hospital, Móstoles, Madrid, Spain; 5 Department of Psychiatry, General Hospital of Villalba, Madrid, Spain; 6 Department of Psychiatry, Infanta Elena University Hospital, Valdemoro, Madrid, Spain; 7 Department of Psychology, Universidad Católica de Maule, Talca, Chile; 8 Department of Psychiatry, Centre Hospitalier Universitaire de Nîmes, Nîmes, France; 9 Centro de Investigación Biomédica en Red de Salud Mental (CIBERSAM), Research Group CB/07/09/0025, Madrid, Spain

**Keywords:** administrative criteria, depression, hospitalization, recurrence, relapse

## Abstract

**Background:**

Major depressive disorder (MDD) is a leading cause of disability globally, with high relapse rates. Currently, there is no standardized consensus on administrative criteria for defining relapses in MDD.

**Methods:**

A comprehensive literature search was conducted for texts published from January 1980 to April 2026. Articles were included if they described a depressive episode/major depressive episode or unipolar depression. The initial electronic search identified 4148 documents, and 51 were finally included in this review.

**Results:**

Over two thirds of the articles focus directly on relapse of MDD, either describing the overall prognosis of the disorder in various populations, the outcome of a given intervention, or the impact of certain factors. 44 of the 51 articles provide a definition of relapse based on either clinical criteria, psychometric measures or what we will establish as administrative criteria. Among those who use scales for definition there is not much uniformity. Among the administrative criteria the most cited is hospitalization, followed by changes in treatment and the need for electroconvulsive therapy.

**Conclusions:**

We suggest a conceptual model based on these criteria to define relapse in depression by drawing some preliminary conclusions: A minimum period of time from the remission of the previous episode to the emergence of what is considered a relapse is necessary, as well as the presence of at least one high-specificity criterion—hospitalization (including emergency room visit), ECT, or suicide attempt—and, in a complementary role, structured pharmacological changes that reflect guideline-based therapeutic steps.

## 1 Introduction

### 1.1 Context and relevance

Major depressive disorder (MDD) is a leading cause of disability globally, with high relapse rates despite advances in treatment. It is estimated that up to 50-80% of patients who have experienced a major depressive episode will experience at least one relapse in their lifetime (1). Patients with a history of relapse have a more complex MDD profile (2), and it is well studied that patients who experience relapses consume a greater amount of health resources, which translates into a higher annual cost both in total and in relation to MDD (1). Also, the more depressive episodes, the higher the probability of relapse (3). Therefore, early identification and appropriate management of relapses are crucial to reducing the burden of illness and improve clinical outcomes.

### 1.2 Definition of relapse and review of the current state

When trying to define the concept of relapse, we often encounter conceptual difficulties (4, 5). In the clinical setting, relapse of MDD is traditionally defined as the reappearance of depressive symptoms within a period of remission, but without reaching a complete recovery, as distinguished from recurrence, which implies a new episode after full recovery (6). However, in health systems and in administrative management, relapses are often evaluated with different criteria, often based on hospital readmissions, use of health resources or adjustments in pharmacotherapy. These discrepancies can create barriers to accessing appropriate treatment and affect the quality of care.

Frank et al. (5) were the first to propose consensus definitions for the terms relapse, recurrence, and remission. Although they propose a definition, it remains based on purely clinical criteria (referencing operational criteria when discussing the duration and severity or intensity of symptoms). To this day, despite this proposal, there is still no temporal definition of relapse, nor is there consensus on administrative criteria to define relapse in depression.

Contemporary research increasingly conceptualizes depression not as a single, discrete disorder but as a heterogeneous, chronic, and dynamically fluctuating syndrome characterized by variable symptomatic trajectories. Longitudinal studies show that many patients experience partial remission, unstable improvement, or recurrent fluctuations rather than clearly demarcated episodes, challenging traditional episodic models of the illness. This evolving understanding has major implications for defining relapse, as rigid symptom−based or episode−based boundaries may fail to capture clinically meaningful deterioration across diverse trajectories. Recognizing this heterogeneity strengthens the rationale for developing pragmatic administrative criteria that can be applied consistently across health−system datasets, regardless of the underlying clinical course.

#### 1.2.1 Conceptual and operational challenges in defining remission

Current evidence indicates that only approximately 35–45% of patients achieve symptomatic remission following antidepressant treatment in real−world settings (56). This low rate has important methodological implications, because any definition of relapse necessarily presupposes a prior state of remission. According to DSM−5, remission requires the absence or near−absence of depressive symptoms for a sustained period; however, in practice, many patients exhibit partial remission, residual symptoms, or fluctuating symptom trajectories, making the boundary between remission and ongoing symptomatic states difficult to operationalize.

These conceptual challenges are amplified in administrative datasets, where remission cannot be directly measured through symptom scales or structured clinical assessments. Instead, remission must be inferred indirectly—typically through the absence of treatment changes, hospitalizations, or emergency presentations—an approach that risks conflating true remission with periods of clinical stability or non−attendance. Recognizing these limitations is essential, as the validity of any operational definition of relapse depends on the robustness and clarity of the remission construct on which it is built.

### 1.3 Justification of the study

Currently, there is no standardized consensus on administrative criteria for defining relapses in MDD. Some health systems use indicators such as the frequency of consultations, changes in antidepressant prescription or time between episodes to define relapse, which may not accurately reflect the patient’s experience. There are studies that define relapse based on interviews with patients by collecting reappearance of symptoms (7–12, 41–43) or passing scales and comparing changes in the score with respect to previous evaluations (4, 7, 13–18, 42–49). This method may lead to overdiagnosis of recurrences. Other studies (1, 3, 19–29, 50–55), however, establish as criteria only factors that are more administrative than clinical, such as the increase in referrals or reinitiation of treatments, which, on the contrary, has the advantage of being purely descriptive but with a risk of underdiagnosing relapses (4). This variability raises questions about the validity and clinical utility of these criteria.

### 1.4 Aim of the study

This article aims to critically analyse the administrative criteria used to define relapses in MDD and discuss their implications for clinical practice and health policy formulation. The current literature will be reviewed and the impact of these criteria on the identification and management of relapses will be evaluated.

## 2 Methods

### 2.1 Literature search strategy

A comprehensive literature search was conducted in the MEDLINE database for records published between January 1980 and April 2026. The search strategy was based on the following MeSH (Medical Subject Headings) terms: Depressive Disorder NOT Bipolar Disorder AND (Relapse OR Recurrence) AND (Hospitalization OR Emergency Room Visits OR Missed Appointments OR Non-Attendance OR No-Shows OR Outpatient Default OR Extra Appointments OR Outpatient Visits). The search was limited to titles and abstracts. The date of the last search was May 2026. Retrieved records were screened according to predefined eligibility criteria.

### 2.2 Inclusion criteria and retrieved articles

Articles were included if they were published in English or Spanish, if they included adult cases, described a depressive episode/major depressive episode or unipolar depression. Samples containing paediatric patients were not included. No studies in which the diagnoses were not performed by specialized mental health personnel were included. Retrospective, and prospective studies were included, as well as clinical trials. We omitted case control studies and studies that included bipolar depression as a diagnosis. Citations within identified articles were included as additional sources.

We omitted case series, uncompleted studies, conference abstracts or doctoral theses. The initial electronic search identified 4148 documents. After evaluating the abstracts, we selected 448 articles that met the criteria, and 51 were included in this review. When selecting the articles, we were aware of unavoidable biases such as selection bias (differences between baseline characteristics of the groups) and detection bias (differences in how outcomes are measured).

### 2.3 Operative definitions

For clarity, we will reference three distinct criteria: purely clinical criteria, which are directly related to the patient’s symptoms or biological responses; clinical criteria based on validated psychometric tools, which incorporate standardized subjective measurements; and administrative criteria, which pertain primarily to operational aspects and are not dependent on clinical characteristics.

While many of the studies focus on clinical or psychometric criteria, most also address operational aspects. We have grouped these into five categories.

- Hospitalization.
- Emergency visit.
- Suicide or suicide attempt.
- Antidepressants. Any change at the pharmacological level, from introduction, to upward adjustments of doses, potentiation or change of psychotropic drugs.
- ECT. Including both cases in which it was necessary to initiate such therapy and those in which it was necessary to reintroduce it after having previously received it.

For studies reporting time-to-relapse estimates, we extracted the median time from remission to the first relapse event when available, or the closest equivalent measure when medians were not explicitly provided. These values were used to derive an empirical temporal threshold.

## 3 Results

In this review, we will briefly describe and compare the studies that have evaluated the administrative/clinical criteria for relapses in MDD in the last 45 years (from 1980 to 2026).

### 3.1 Study characteristics

Of the total of 51 articles published between 1980 and 2026, 37 are from the last 15 years, that is, 72,5%. The samples with the highest number of patients (>10.000) are in South Korea (22, 26), Germany (30), UK (55) and the US (1, 31, 32). There are samples of very extreme N (up to 752.190 patients), with yet a median for all studies of 135.5. Half of the studies are carried out with patient samples obtained in an outpatient setting (1, 8, 10, 15–22, 24, 26, 31–37, 42, 44, 45, 48, 53, 55) and the other half in a hospital setting (9–14, 16, 23, 25, 27, 29, 30, 38–40, 46, 50–52, 54). Three articles include patients from both fields (4, 41, 47) and in two the setting is not reported and there are no data from which this can be inferred (43, 44). 39,2% of the articles were prospective descriptive (20 of 51), 33.3% were clinical trials (17 of 51), and 27.4% (14 of 51) were retrospective descriptive. The country with the most articles was the US with a 23,5% of the total (12 of 51), followed by the UK with a 19.6% (10 of 51). The characteristics of the included studies are summarized in **Table 1**.

**Table 1:** Summary of included studies and their main characteristics.

	Year	Country	Setting	N	Time of study (years)	Type of study	Criteria
Aparicio L VM, et al (44)	2026	Brasil	Outpatients	71	0.46	Descriptive prospective	Hospitalization Suicide or suicide attempt Other serious clinical events requiring treatment modification
Tonon AC, et al (45)	2026	Canada	Outpatients	94	1	Descriptive prospective	Hospitalization Suicide or suicide attempt AD (escalation)
Lisha I, et al (50)	2026	US	Inpatients	159	2	Descriptive retrospective	Hospitalization Suicide or suicide attempt AD (change) Necessitiy of ECT Requirement for additional neuromodulation interventions
Del Casale A, et al. (41)	2025	Multinational	Both	55.480	5	Descriptive retrospective	Hospitalization Suicide or suicide attempt
Cheng YC, et al (42)	2025	Taiwan	Outpatients	39	2	Clinical trial	Hospitalization AD (change)
Van Diermen L, et al (46)	2025	Belgium	Inpatients	65	0.5	Descriptive prospective	Restart of ECT Suicide or suicide attempt
Hao X, et al (51)	2025	China	Inpatients	3977	2	Descriptive retrospective	Hospitalization
Brakemeier EL, et al	2025	Germany	Both	396	1.3	Clinical trial	Hospitalization
Kronsell A, et al (52)	2025	Sweden	Inpatients	2858	1	Descriptive retrospective	Hospitalization Suicide or suicide attempt Restart of ECT
Lam R, et al. (48)	2025	Canada	Outpatients	96	2	Descriptive prospective	Hospitalization Suicide or suicide attempt AD (iniciation or change)
Li F, et al (53)	2025	Netherlands	Outpatients	6863	10	Descriptive retrospective	New treatment episode following a 6-month service-free
Young A, et al (43)	2024	Multinational	NA	676	0.66	Clinical trial	Hospital admission Suicide or suicide attempt
Kim H, et al (49)	2024	USA & Canada	NA	126	1	Clinical trial	Hospitalization Suicide or suicide attempt
Yao Y, et al (54)	2024	China	Inpatients	6113	16	Descriptive retrospective	Hospitalization
Cao Z, et al (55)	2024	UK	Outpatients (General Population)	26164	13.3	Descriptive prospective	Hospitalization
Lambrichts S, et al (19)	2022	Netherlands	Outpatients	110	5	Descriptive prospective	Hospital admission Necessity of ECT Suicide or suicide attempt
Touya M, et al (1)	2022	US	Outpatients	14093	7.5	Descriptive retrospective	Hospitalization Necessity of ECT Suicide or suicide attempt AD (reinitiation) Emergency visit
Kofod J et al (20)	2022	Denmark	Outpatients	90	10	Clinical trial	Hospital contacts
Okereke OI, et al (31)	2021	US	Outpatients (General population)	18353	5.3	Clinical trial	AD (+-counseling)
Appleton KM, et al (7)	2021	UK	Review	35 studies 1964 patients	Review	Review of clinical trials	Hospitalization
Powell V, et al (35)	2021	UK	Outpatients	148	13	Descriptive retrospective	Hospitalization
Van de Velde N, et al (21)	2021	Belgium	Outpatients	37	0,25	Descriptive prospective	Hospitalization Necessity of ECT
Okereke OI, et al (32)	2020	US	Outpatients	18353	3,6	Clinical trial	AD (+- counceling)
Cabelguen C, et al (36)	2020	France	Outpatients	16	5.1	Descriptive retrospective	Hospitalization
Lin CH, et al (13)	2020	Taiwan	Inpatients	97	5.5	Clinical trial	Hospitalization
Yang, W. C., et al (14)	2020	Taiwan	Inpatients	130	5.5	Clinical trial	Hospitalization
Kim, M. J.,et al (22)	2019	South Korea	Outpatients (General population)	752190	1.5	Descriptive prospective	AD (re-initiation)
Durgam, S.,et al (15)	2019	US	Outpatients	274	1	Clinical trial	Hospitalization Suicide or attepted suicide AD (change)
Wiegand, H. F., et al (30)	2017	Germany	Inpatiens	58913	1	Descriptive prospective	Hospitalization
Rosen, B. H., et al (23)	2016	US	Inpatients	482	5	Descriptive Retrospective	Hospitalization
Verwijk, E., et al (16)	2015	Netherlands	Inpatients	87	0.5	Clinical trial	Hospitalization Necessity of ECT Suicide of suicide attempt
Rosenberg, O., et al (17)	2015	Israel	Outpatients	17	0.77	Descriptive prospective	Hospitalization Necessity of ECT or any other neuro-stimulation treatment AD (switching, augmentation, addition, and dosage escalation)
Atiku, L., et al (24)	2015	UK	Outpatients	102	0.5	Descriptive retrospective	Hospitalization AD (review)
Rosenthal, J. Z.,et al (18)	2013	US	Outpatients	874	1	Clinical trial	Hospitalization Suicide or suicide attempt
Hansen, H. V., et al (25)	2012	Denmark	Inpatients	268	1	Clinical trial	Hospitalization
Moksnes K. M (8)	2011	Norway	Outpatients	141	35	Descriptive reprospective	Hospitalization Necessity of ECT AD (change)
Kim, K. H., et al (26)	2011	South Korea	Outpatients	117087	3	Descriptive retrospective	Hospitalization Necessity of ECT Suicide or suicide attempt AD Emergency visit
Goodwin, G. M., et al (37)	2009	Australia, Finland, France, UK & South Africa	Outpatients	339	2	Clinical trial	Suicide or suicide attempt Withdrawal for lack of efficacy
Akerblad, A. C., et al	2006	Sweden	Outpatients	835	2	Descriptive prospective	Hospitalization Suicide or suicide attempt AD
Prudic, J., et al (38)	2004	US	Inpatients	398	0.5	Descriptive prospective	Hospitalization Necessity of ECT Suicide or suicide attempt Psychotic symptoms
Kessing L. V. (39)	2003	Denmark	Inpatients	3455	6	Descriptive prospective	Hospitalization
Wilkinson, D., et al (40)	2002	UK	Inpatients	49	2	Clinical trial	Necessity of ECT AD (increase or change)
Mundt, C., et al (9)	2000	Germany	Inpatients	76	2	Descriptive prospective	Hospitalization
O’Leary, D., et al (10)	2000	Ireland	Inpatients	100	1.5	Descriptive prospective	AD (increase)
Melfi, C. A., et al (27)	1998	US	Inpatients	4052	2	Descriptive prospective	Hospitalization Necessity of ECT Suicide or suicide attempt AD Emergency visit
O’Leary, D. A.,et al (28)	1996	UK	Outpatients	96	7	Descriptive prospective	Hospitalization
Kocsis, J. H., et al (34)	1996	US	Outpatients	129	2	Clinical trial	AD (change)
Colenda, C. C., et al (29)	1991	US	Inpatients	111	2	Descriptive retrospective	Hospitalization
Hooley, J. M., et al (11)	1989	UK	Inpatients	39	0.75	Descriptive prospective	Hospitalization
Hooley, J. M., et al (12)	1986	UK	Inpatients	39	0.75	Descriptive prospective	Hospitalization
Blackburn, I. M., et al (4)	1986	UK	Both	41	2	Descriptive prospective	Hospitalization AD (reinitiation)

### 3.2 Main aspects addressed

More than half of the articles focus directly on relapse of MDD, either describing the overall prognosis of the disorder in various populations, the outcome of a given intervention, or the impact of certain factors. More than half (58.8%) of the articles study the impact of a given intervention (30 of 51). Other studies evaluate the influence on the evolution of MDD of the existence of other concomitant mental disorders, such as ADHD (35) or a personality disorder (30).

### 3.3 Definitions provided for the concept of relapse

44 of the 51 articles (86.3%) provide a definition of relapse based on one or more criteria. Some of them establish a difference between the terms “relapse” and “recurrence” (4, 28). 9 of the articles that give a definition of relapse do so based on clinical criteria (DSM III, DSM IV, reappearance of depressive symptoms) (7–12, 41–43), 20 do so with psychometric measures (Hamilton Depression Rating Scale, Beck Depression Inventory, Montgomery-Asberg Depression Rating Scale, Goldberg Anxiety and Depression Scale, Clinical Global Impression) (4, 7, 9, 13–18, 33, 34, 42–49) and 40 define it based on what we will establish as administrative criteria (1, 4, 7, 9, 11–13, 15–29, 33, 34, 38, 39, 42–50, 52–55).

Among those who use scales for definition, there is not much uniformity in the criteria. 13 of the 20 use the Hamilton Depression Rating Scale, although the score they establish as a criterion is very varied, with some considering a score of 8 points (4) as sufficient for a relapse, others of 12 (20), 14 (13, 14, 44), 16 (17, 18) 18 (47, 49) and up to 20 (9). The second most used scale is the Montgomery-Asberg Depression Rating Scale (MADRS), with 7 studies establishing the definition of relapse based on it, although again the criteria vary; for Verwijk et al. (16) and van Diermen et al. (46) a score of 15 or more would be sufficient, for Durgam et al. (15), 18 or more, for Åkerblad et al. (33) up to 21 points or an increase of 50% with respect to previous assessments would be needed, and for Young et al., 2024 (43), Tonon et al., 2026 (45) and Lam et al., 2025 (48) a score greater than or equal to 22 for 2 consecutive weeks. Finally, 4 studies use the Clinical Global Impression (CGI-S) (15, 33, 43, 48), 2 use the Beck`s Depression Inventory (4, 9) and 1 uses the Goldberg Anxiety and Depression Scale (GADS) (20).

### 3.4 Administrative criteria

Regarding the administrative criteria that have been established, the one mentioned in the largest number of articles is hospitalization, being mentioned in 42 of the 51 (82,3%) articles included in the review (1, 2, 4–7, 9–17, 19–21, 23–31, 34, 35, 41–45, 47–52, 54, 55). The second criterion with which most authors agree is that related to antidepressant treatment, although some mention it in general (27, 33) and others specify whether they consider a change of antidepressant (8, 15, 26, 34, 40, 42, 48, 50), an upward adjustment (10, 17, 40, 45), a review of the treatment (24) or a potentiation (17). Two articles mention antidepressant treatment with or without combination with psychotherapy (31, 32). Suicide/suicide attempt is mentioned 19 times (1, 15, 16, 18, 19, 26, 27, 33, 37, 38, 41, 43–46, 48–50, 52) and necessity of ECT 13 times (1, 8, 16, 17, 19, 21, 26, 27, 38, 40, 46, 48, 50, 52). Finally, 3 articles mention Emergency visit as a possible indicator of relapse (1, 26, 27).

### 3.5 Time−to−relapse data and derivation of the temporal threshold

Fourteen studies in the review provided extractable quantitative estimates of time to relapse. For each study, we recorded the reported median time from remission to the first relapse event (or the closest available estimate when medians were not explicitly provided), ensuring that all values reflected the interval between remission and the first clinically defined relapse event. The extracted medians showed substantial heterogeneity, ranging from very early relapse in severe or treatment−resistant samples with medians between 53 and 90 days (14, 21, 23, 38) to substantially longer intervals in maintenance−treated populations (218–294 days) (33, 40, 45).

To obtain a pragmatic estimate applicable across heterogeneous clinical contexts, we calculated the median of the study−level medians. This approach reduces the influence of extreme values and reflects the central tendency across the available evidence. The resulting value was approximately 90 days, indicating that half of the studies reporting time to relapse observed relapse events within the first three months after remission.

This empirical estimate served as the basis for proposing a minimum operational interval between remission and the emergence of administrative criteria indicative of relapse.

## 4 Discussion

While this review was initially conceived as the first comprehensive proposal of administrative operational criteria for defining relapse in MDD, a recent study by Lisha et al. (2026) has independently advanced a conceptually related approach. In a longitudinal cohort of Veterans with treatment-resistant depression treated with TMS, the authors introduced the SPEAR (Significant Psychiatric Events Associated with Relapse) composite outcome, which defines relapse exclusively through high-severity administrative events—psychiatric hospitalization, suicide attempt, death by suicide, or the need for additional neuromodulation (ECT or a new TMS course). SPEAR was used as the primary endpoint in their survival analyses and was explicitly proposed as a pragmatic alternative to symptom-based definitions, particularly in settings where routine measurement-based assessments are not available. Their findings illustrate the feasibility of using administrative events as markers of relapse, while also highlighting limitations such as the heterogeneity of the composite, the predominance of repeat TMS as the main driver of events, and the potential for artificially extended durability estimates due to the severity threshold required for SPEAR events.

However, SPEAR focuses exclusively on high-severity events, whereas the present review aims to develop a broader operational framework applicable across heterogeneous clinical settings.

In addition, recent regulatory developments reinforce the need for clear and operational relapse definitions. The EMA guideline on the clinical investigation of medicinal products for depression (EMA/CHMP/185423/2010 Rev. 3, adopted January 2025) provides updated recommendations on the operationalization of relapse endpoints in maintenance trials, underscoring the importance of standardized and reproducible criteria. These regulatory considerations align with the broader administrative framework proposed in this review.

The empirical synthesis of time-to-relapse data (Section 3.5) provides a necessary foundation for interpreting the temporal dimension of relapse definitions.

A further issue concerns the clinical and methodological relevance of establishing standardized administrative indicators. Administrative events are consistently recorded, less susceptible to subjective variability, and allow population−level monitoring across health systems. Their standardization would facilitate comparability across studies, support service planning, and complement rather than replace clinical assessments. At the same time, clinical interviews and symptom scales may contribute to overdiagnosis by capturing transient fluctuations, measurement noise, or non−clinically significant changes. Conversely, administrative criteria carry the opposite risk: they may underdiagnose relapse by preferentially identifying severe, highly recurrent, or treatment−resistant cases while missing milder deteriorations. A balanced framework must therefore acknowledge both sources of bias and integrate them into a coherent operational definition.

### 4.1 A minimum period from the remission of the previous episode to the emergence of what is considered a relapse

The reviewed literature shows that, although numerous studies report time−to−relapse using Kaplan–Meier curves, none proposes an explicit temporal threshold that allows an operational distinction between relapse and other clinical or administrative events. This absence of consensus is particularly relevant for health systems, where relapse definitions must be operational, reproducible, and applicable to large populations.

The studies that do provide quantifiable time−to−relapse estimates show considerable heterogeneity, driven by differences in population, severity, treatment modality, and follow−up duration. Despite this variability, a consistent pattern emerges: clinically meaningful relapse events tend to cluster within the first months following remission, particularly among patients with greater baseline severity or treatment resistance. Very early symptom fluctuations are therefore more likely to reflect incomplete remission or residual symptomatology rather than true relapse.

From an operational standpoint, a minimum interval of approximately 90 days provides a pragmatic threshold that reduces misclassification, aligns with typical follow−up cycles in routine care, and captures the period in which relapse risk is empirically highest. Importantly, this threshold should not be interpreted as a universal marker of the natural course of depression, but rather as a practical criterion that enhances the consistency and applicability of administrative definitions of relapse across health−system contexts.

The temporal dynamics of depressive episodes also influence whether clinical worsening should be interpreted as relapse or as part of a fluctuating symptomatic trajectory. Short or unstable remission periods increase the likelihood that early symptom returns reflect incomplete recovery, whereas longer periods of stability make subsequent deterioration more consistent with true relapse. These dependencies highlight that relapse is embedded within longitudinal patterns and reinforce the need for operational criteria that incorporate both temporal thresholds and clinical context.

### 4.2 Establishing the importance of each administrative criterion described and the necessity of their presence to consider an episode as a relapse

Across the reviewed literature, the administrative criteria most frequently used to define relapse events include hospitalization, changes in pharmacological treatment, and the need for electroconvulsive therapy (ECT). These criteria appear consistently across studies spanning several decades, reflecting their clinical relevance and their feasibility for use in large-scale health-system datasets.

Hospitalization is the most commonly cited administrative marker. In this review, we considered hospitalization to include both inpatient admissions and emergency room visits, as the few studies that incorporate emergency presentations also include inpatient hospitalization within the same criterion. Although some authors equate relapse with rehospitalization—treating readmission rates as equivalent to relapse rates (20, 28)—others report these outcomes separately. For example, Hooley and Teasdale (11) reported relapse rates of 51% and readmission rates of 35%, demonstrating that these constructs do not fully overlap. Nevertheless, multiple studies have shown a statistically significant association between relapse and hospitalization or emergency presentations (1). As Rosen et al. (23) notes, hospitalization may underestimate the true relapse rate, but it remains a clinically and administratively meaningful indicator of deterioration and a useful outcome for evaluating treatment effectiveness. Similarly, Colenda et al. (29) used hospitalization as the primary outcome measure, acknowledging that not all relapses require admission but that hospitalization reliably captures severe clinical worsening. Prudic et al. (38) and Atiku et al. (24) likewise consider hospitalization, ECT, treatment reintroduction, or suicide attempts sufficient to define relapse.

Pharmacological treatment modifications are frequently used as administrative indicators of relapse, but they require careful interpretation. Many adjustments reflect partial response, residual symptoms, tolerability issues, or routine optimization rather than the onset of a new depressive episode. For this reason, medication changes should be considered supportive indicators rather than primary markers.

To increase specificity, only structured treatment modifications aligned with major clinical guidelines should be included. NICE NG222, the APA Practice Guideline, and the Spanish National Health System Guideline identify three types of clinically meaningful therapeutic steps:

- dose escalation within the therapeutic range when improvement is insufficient,
- switching antidepressants after inadequate response,
- augmentation strategies such as lithium or atypical antipsychotics.

These guideline-based interventions reflect deliberate clinical decisions made in response to significant deterioration and therefore provide a more reliable operational signal than minor or routine medication adjustments.

## 5 Conclusions

Based on our literature review, we tentatively draw some preliminary conclusions:

A minimum period of 90 days from remission is necessary, as well as the presence of at least one high-specificity criterion—hospitalization (including emergency room visit), ECT, or suicide attempt—and, in a complementary role, structured pharmacological changes that reflect guideline-based therapeutic steps.

These criteria should be viewed as a preliminary operational framework that requires prospective validation across diverse clinical settings and health-system structures.

## 6 Limitations

While this proposal is grounded in evidence drawn from previous studies, it should be regarded as an initial approximation. The search covered an extended time frame, which implies that changes in diagnostic criteria, clinical practices, and social contexts over time may have influenced the comparability among studies, although this issue was not explicitly analysed. Also, the selected studies exhibit substantial heterogeneity, which hampers direct comparison between them and limits the potential to draw generalizable conclusions. Further research is essential to evaluate the sensitivity, specificity, and applicability of this approach across different populations and healthcare systems.

An additional limitation concerns the inclusion of suicide attempts as an administrative marker of relapse. Although suicide attempts have been consistently used across decades of research—and are incorporated into composite outcomes such as SPEAR—they lack diagnostic specificity. Suicidal behaviour may arise in the context of personality disorders, substance use, acute psychosocial crises, or adjustment disorders, and therefore cannot be assumed to reflect a depressive relapse in all cases. For this reason, suicide attempts are retained in our framework as high-severity indicators, but their interpretation must be contextualized within the broader clinical picture and not treated as a standalone marker of depressive relapse.

Furthermore, the derivation of the temporal threshold was limited to the subset of studies reporting extractable time-to-relapse estimates, which may introduce bias and restrict generalizability.

A further limitation concerns the difficulty of distinguishing relapse from symptom persistence or fluctuation in chronic or unstable depressive trajectories. Because many patients experience residual symptoms or incomplete remission, administrative indicators may capture episodes of worsening that do not correspond to a discrete relapse. This ambiguity underscores the need for future work integrating administrative markers with longitudinal symptom data.

## Data Availability

The original contributions presented in the study are included in the article/supplementary material. Further inquiries can be directed to the corresponding author.
